# Benchmarking Donor Safety: Postoperative Complications and Risk Stratification in 502 Living Liver Donors

**DOI:** 10.3390/medicina62020358

**Published:** 2026-02-11

**Authors:** Adem Tuncer, Emrah Sahin, Bulent Unal, Abuzer Dirican

**Affiliations:** Department of General Surgery, VM Medical Park Florya Hospital, Istanbul Aydin University, 34295 Istanbul, Turkey; dr.emrahsahin@gmail.com (E.S.); bdozelsaglik@gmail.com (B.U.); abuzerdirican@hotmail.com (A.D.)

**Keywords:** donor safety, hepatectomy, postoperative complications, Clavien–Dindo classification, body mass index

## Abstract

*Background and Objectives*: Living donor hepatectomy is an essential component of liver transplantation programs, with donor safety representing the foremost priority. This study aimed to evaluate early postoperative complications in living liver donors and to identify clinical and demographic factors associated with complication risk using the Clavien–Dindo classification. *Materials and Methods*: A retrospective analysis was conducted on 502 consecutive living liver donors who underwent hepatectomy between August 2021 and May 2025. Donors received standardized preoperative evaluation, surgical management, and postoperative follow-up. Demographic characteristics, graft-related variables, remnant liver ratio, and clinical outcomes were recorded. Postoperative complications were graded using the Clavien–Dindo classification, with Grade ≥ IIIa defined as major complications. Univariable and multivariable logistic regression analyses were performed. *Results:* Postoperative complications occurred in 58 donors (11.6%; 95% CI: 9.0–14.6%), the majority of which were mild to moderate (Grades I and II). Biliary complications were the most frequent cause of morbidity. Major complications (≥Grade IIIa) were observed in 17 donors, while no Grade IV and V complications or mortalities were recorded. Donors with complications had significantly longer hospital stays (*p* = 0.0002). Although crude complication rates were higher among Turkish donors than foreign donors (13.9% vs. 7.5%, *p* = 0.043), this association did not remain statistically significant after multivariable adjustment. No independent associations were identified between complication risk and graft type, remnant liver ratio, graft volume, or BMI. *Conclusions:* Living donor hepatectomy was associated with a low rate of severe early postoperative complications under standardized protocols. However, given the retrospective design and limited structured long-term follow-up, these findings primarily reflect early postoperative safety. Biliary complications remain the most common postoperative issue. Further multicenter prospective studies with extended follow-up are needed to comprehensively assess long-term donor outcomes.

## 1. Introduction

End-stage liver disease (ESLD) represents a critical global health challenge, necessitating liver transplantation (LT) as the definitive and often sole life-saving therapeutic intervention for patients facing irreversible organ damage [1]. However, the persistent mismatch between the growing demand for transplantation and the limited availability of deceased donor organs continues to result in substantial waiting-list mortality worldwide [2]. In response to this critical shortage, living donor liver transplantation (LDLT) emerged as an alternative strategy in the late 1980s and has since become increasingly adopted, particularly in regions with low rates of deceased organ donation [3,4]. LDLT plays a pivotal role in expanding the available organ pool, thereby significantly improving recipient survival rates and effectively reducing mortality associated with prolonged waiting periods [5,6]. Recognizing these profound advantages, the number of centers worldwide performing living donor transplants has steadily increased [7].

Despite these advantages, LDLT presents a unique ethical and clinical challenge: a healthy individual voluntarily undergoes major elective surgery for the benefit of another. Therefore, donor safety is not merely a clinical goal but the central principle governing all LDLT programs [8]. International clinical practice guidelines, including the most recent European Association for the Study of the Liver (EASL) Clinical Practice Guidelines on Liver Transplantation (2024) and the American Association for the Study of Liver Diseases (AASLD)/American Society of Transplantation (AST) Practice Guidance (2025), underscore the critical role of systematic complication reporting, continuous outcome surveillance, and structured long-term donor follow-up as integral pillars of donor safety [9,10].

Although living donor hepatectomy is generally considered safe, it is not devoid of risk. A recent meta-analysis involving 60,829 living liver donors reported an overall morbidity rate of 24.3% and a mortality rate of 0.06% [11]. These figures underscore that while living donor hepatectomy is generally safe, it carries a discernible risk of complications. In this context, it is critically important for transplantation centers to routinely and systematically analyze their donor surgical outcomes using standardized classification systems, such as the Clavien–Dindo system [12,13]. Previous studies employing this system have consistently identified biliary complications—particularly bile leakage and biliary strictures—as the most common sources of postoperative morbidity, frequently exceeding 10% [14]. Other reported complications include wound-related problems, pleural effusion, ileus, infections, pulmonary events, cardiovascular complications, and, rarely, mortality [15,16].

Most postoperative complications in living liver donors occur during the early postoperative period and are typically mild to moderate in severity, including pleural effusion, wound-related issues, and transient medical complications [17]. Therefore, short-term follow-up can provide valuable insight into early donor safety. Nevertheless, long-term follow-up remains essential for detecting late-onset complications, low-frequency adverse events, and psychosocial outcomes that may not be captured in early postoperative surveillance.

In this context, the present study aimed to benchmark donor safety through a comprehensive retrospective analysis of 502 consecutive living liver donor hepatectomies performed at a single center over a 3,5-year period. The primary objective was to evaluate the incidence and severity of postoperative complications using the Clavien–Dindo classification system. Secondary objectives included the identification and risk stratification of demographic and clinical factors potentially associated with postoperative morbidity.

## 2. Materials and Methods

### 2.1. Study Design and Patient Selection

This retrospective cohort study included 502 consecutive living donors who underwent hepatectomy for liver transplantation at our institution between August 2021 and May 2025. Ethical approval was obtained from the Istanbul Aydın University Non-Interventional Clinical Research Ethics Committee (No: 152/2025). To minimize selection bias, all consecutive donors during the study period were included in the analysis.

### 2.2. Preoperative Evaluation

All prospective donors underwent a comprehensive preoperative assessment to ensure medical, surgical, and psychosocial suitability for donation. This evaluation included a detailed physical examination, complete blood count, biochemical profile, liver function tests, and coagulation studies, including screening for Factor V Leiden mutation and prothrombin gene variants. Viral serologies, urine cultures, and blood group typing were routinely performed.

Triphasic computed tomography (CT) was used for volumetric analysis, assessment of hepatic steatosis, and evaluation of vascular anatomy. Donors who passed the initial screening were further assessed by cardiology, pulmonology, gastroenterology, and psychiatry specialists as part of a multidisciplinary evaluation process.

### 2.3. Donor Acceptance Criteria

Donor selection was guided by strict safety-oriented principles. Individuals with moderate-to-severe hepatic steatosis were excluded due to the well-documented association between steatosis, impaired liver regeneration, increased postoperative morbidity, and compromised donor safety. Donors with mild steatosis were considered eligible only if an adequate remnant liver volume could be preserved, in accordance with previously reported extended donor safety frameworks [18].

### 2.4. Data Collection and Statistical Analysis

Demographic data (age, sex, height, weight, body mass index [BMI], and nationality) and surgical variables (graft type, resected graft volume, and remnant liver ratio) were recorded. Clinical outcomes, including the type and grade of postoperative complications and length of hospital stay (LOS), were retrieved from the institutional electronic medical record system.

BMI was calculated as weight in kilograms divided by height in meters squared (kg/m^2^) and categorized into three groups: <20, 21–29, and ≥30. These categories were not based on the standard World Health Organization classification but were defined according to clinically relevant surgical risk thresholds. Donors with a BMI < 20 kg/m^2^ were analyzed separately due to concerns regarding reduced physiological reserves and potential malnutrition, which may adversely affect postoperative recovery [19]. Similarly, donors with BMI ≥ 30 kg/m^2^ were grouped separately because obesity has been associated with increased perioperative risk, impaired wound healing, and higher complication rates [20,21]. Donors with BMI values between 21 and 29 kg/m^2^ represent the range most commonly accepted by transplant centers for living donation.

Graft weight was measured intraoperatively using a calibrated precision balance. All postoperative complications and their corresponding therapeutic interventions were systematically documented. Complication severity was retrospectively graded according to the Clavien–Dindo classification through consensus among four authors to enhance inter-rater reliability. This system ranks complications primarily according to the therapy required to treat the event rather than the diagnosis itself. Grade I includes any deviation from the normal postoperative course without the need for pharmacological treatment or surgical, endoscopic, or radiologic interventions (with permitted supportive medications such as antiemetics, antipyretics, analgesics, diuretics, electrolytes, physiotherapy, and bedside wound opening/drainage). Grade II requires pharmacological treatment beyond these measures and includes blood transfusion and total parenteral nutrition. Grade III comprises complications requiring surgical, endoscopic, or radiologic intervention (IIIa without general anesthesia; IIIb under general anesthesia). Grade IV denotes life-threatening complications requiring ICU management (IVa single-organ dysfunction; IVb multi-organ dysfunction), and Grade V denotes death. Accordingly, identical clinical entities may be assigned to different grades depending on treatment intensity. Major complications were defined as Clavien–Dindo grade ≥ IIIa [12].

Categorical variables were compared using the chi-square (χ^2^) test, and continuous variables were analyzed using the Mann–Whitney U test. A *p*-value < 0.05 was considered statistically significant. Statistical analyses were performed using IBM SPSS Statistics, version [25.0] (IBM Corp., Armonk, NY, USA).

In addition to univariable analyses, a multivariable logistic regression model was constructed to identify independent predictors of postoperative complications. The dependent variable was defined as the presence of any postoperative complication (Clavien–Dindo Grades I–IV). Independent variables included age, sex, BMI, remnant liver volume, graft volume, and donor nationality. Adjusted odds ratios (aORs) with 95% confidence intervals (CIs) were calculated.

### 2.5. Surgical Technique

All donor hepatectomies were performed using an open approach under general anesthesia by the same experienced surgical team. Standard mobilization of the liver was followed by routine cholecystectomy and intraoperative cholangiography. Hilar dissection was performed according to graft type. Parenchymal transection was carried out using ultrasonic aspiration. After division of the vascular and biliary structures, donor stumps were securely closed, and bile leakage was routinely tested. Hemostasis was achieved, a drain was placed, and the abdomen was closed in standard fashion.

### 2.6. Postoperative Management

All donors were managed according to a standardized postoperative care protocol. Patients were routinely monitored in the intensive care unit for at least 24 h before transfer to the surgical ward. Hemodynamic parameters, urine output, and laboratory values—including liver function tests and coagulation profiles—were assessed daily. Pharmacological thromboprophylaxis and early mobilization were initiated in all donors. Abdominal drains were removed based on output volume, biochemical analysis, and clinical findings. Additional imaging studies were performed only in the presence of suspected complications. Routine outpatient follow-up was scheduled, with intensified surveillance for donors who developed postoperative morbidity.

## 3. Results

A total of 502 living donors were included in the analysis. The donor cohort was predominantly young and non-obese, with a relatively narrow distribution of age and BMI values. Donor age was not significantly associated with the development of postoperative complications. In contrast, donors who experienced complications had a significantly longer length of hospital stay, reflecting the clinical burden of postoperative morbidity. Although Turkish donors exhibited slightly higher BMI values compared with foreign donors, BMI itself was not independently associated with complication risk (Table 1).

Postoperative complications were observed in 58 donors, corresponding to an overall complication rate of 11.6% (95% CI: 9.0–14.6%). The most frequently encountered complications included wound problems (*n* = 12), incisional hernia (*n* = 6), meteorism-subileus-ileus (*n* = 6), bile leakage (total *n* = 10), and pleural effusion (*n* = 3). Less common complications observed included atelectasis, pulmonary embolism, intra-abdominal collection, gastritis, and various signs of infection (e.g., urinary tract infection, diarrhea, stool vancomycin-resistant enterococcus (VRE) positivity, etc.) (Table 2).

According to the Clavien–Dindo classification, the majority of complications were of mild to moderate severity (Grades I and II), whereas severe complications (Grade ≥ III) were relatively uncommon. Management strategies varied according to complication severity. Medical treatment alone was sufficient in 29 cases, prolonged drain retention was required in 6 cases, interventional procedures—such as endoscopic retrograde cholangiopancreatography (ERCP), percutaneous transhepatic cholangiography (PTC), or pleurocentesis—were performed in 9 donors, and incisional hernia repair was required in 6 donors. Among donors with bile leakage, percutaneous catheter drainage (PCD) was performed in three cases, ERCP in one case, and a combination of both in four cases. Notably, no re-laparotomy was required for any complication other than incisional hernia repair (Table 2). Importantly, no Grade IV or V complications were observed.

When postoperative complication rates were evaluated across BMI categories, the <20 kg/m^2^ group had a complication rate of 9.1%, the 20–29 kg/m^2^ group 12.6%, and the ≥30 kg/m^2^ group 6.5%. These differences were not statistically significant (*p* = 0.338).

Complications occurred in 44 of 316 Turkish donors (13.9%, 95% CI: 10.5–18.2%) and in 14 of 186 foreign donors (7.5%, 95% CI: 4.5–12.2%). This difference was statistically significant in unadjusted analyses (*p* = 0.043). Regarding severity, complications among Turkish donors were more frequently classified as Grade 2 (*n* = 20) and Grade 3A (*n* = 12), whereas foreign donors predominantly experienced Grade 1 (*n* = 5) and Grade 2 (*n* = 6) complications. However, after adjustment for potential confounders, donor nationality was no longer independently associated with postoperative complication risk.

Complications were observed in 49 of 407 right lobe grafts, 4 of 46 left lobe grafts, and 5 of 49 left lateral grafts, with no significant association between graft type and complication incidence (*p* = 0.759). Similarly, resected graft volume was not significantly associated with postoperative complications (*p* = 0.357). While graft volume categories were defined empirically for descriptive purposes, clinically recognized remnant liver volume thresholds have been established and are closely linked to hepatic parenchymal quality. These thresholds have been well established in the surgical literature and form the basis of modern donor safety paradigms. Previous studies have suggested that a remnant liver volume of at least 20% is acceptable for donors with normal liver parenchyma, whereas higher thresholds—approximately 30% for chemotherapy-affected livers and 40% for fibrotic or cirrhotic livers—are recommended to ensure donor safety [22].

Data on remnant liver ratios were missing for three donors, and graft volume data were unavailable for one donor; therefore, analyses involving these variables were performed using available cases only. When remnant liver ratios were analyzed across predefined categories (<30%, 30–39%, 40–49%, and ≥50%), no statistically significant differences in complication rates were observed (*p* = 0.865). Likewise, no significant association was found between graft volume categories (<500 mL, 500–799 mL, 800–999 mL, ≥1000 mL) and complication development (*p* = 0.702) (Table 3).

In the multivariable logistic regression model, none of the evaluated variables—including age, sex, BMI category, graft volume, remnant liver ratio, and nationality—emerged as independent predictors of postoperative morbidity. These findings indicate that the observed early complications likely reflect multifactorial and procedure-related factors rather than specific donor- or graft-related characteristics (Table 4).

Subgroup analyses were performed to explore potential associations between donor characteristics and complication patterns. However, due to the relatively low number of events, these analyses were underpowered and should be interpreted with caution. The absence of statistically significant associations in these subgroups does not exclude clinically relevant effects but rather reflects limited statistical power.

## 4. Discussion

This study provides a comprehensive evaluation of early postoperative outcomes in a large cohort of 502 consecutive living liver donors. Our findings indicate that living donor hepatectomy can be performed with an acceptable safety profile when normal donor selection criteria, standardized surgical techniques, and structured perioperative care protocols are applied. The overall complication rate of 11.6% observed in our cohort falls within the range reported in previous large series and meta-analyses [23,24]. Importantly, the majority of complications were mild to moderate in severity, and no life-threatening events or donor mortality were recorded.

Consistent with previous reports, biliary complications constituted the most frequent source of postoperative morbidity in our cohort [25,26,27,28]. This observation reflects the inherent vulnerability of the biliary system during donor hepatectomy, particularly in the context of anatomical variations, complex transection planes, and small-caliber bile ducts. Although biliary complications are rarely fatal, they are associated with prolonged hospitalization, increased healthcare utilization, and reduced short-term quality of life. Several studies have emphasized that most biliary complications can be effectively managed using minimally invasive approaches, including endoscopic or percutaneous interventions, thereby limiting the need for reoperation [26,27].

The overall morbidity profile observed in our cohort aligns with the contemporary literature suggesting that early postoperative complications in living donors are predominantly low-grade and transient [23,25]. These findings reinforce the notion that short-term donor safety can be reliably assessed through standardized early postoperative surveillance. However, it should be emphasized that early outcomes alone do not fully capture the long-term physical, psychological, and social consequences of donation, which remain critical components of comprehensive donor safety assessment [24].

LDLT represents a crucial therapeutic option in regions with limited access to deceased donor organs. However, the morbidity and potential mortality associated with donor hepatectomy remain the most critical concerns. To maximize donor safety and minimize adverse outcomes, it is essential to identify and understand potential risk factors. These include donor-related variables such as age, sex, body mass index (BMI), graft type (e.g., right lobe, left lobe, or left lateral segment), and remnant liver volume.

Reported complication rates following living donor hepatectomy vary widely in the literature, typically ranging from 10% to 31%. A large-scale meta-analysis reported an overall complication rate of 24.7%, a major complication rate of 5.5%, and a mortality rate of 0.06% [1,7,23]. In comparison, the overall complication rate observed in our cohort was 11.55%, which is lower than some previously reported rates, such as the 18.6% reported by Ozgor et al. [2], while remaining comparable to others, including the 7.7% reported by Khalid et al. in a cohort of 207 donors [13]. Although our overall complication rate of 11.6% falls within the acceptable range proposed by the AASLD (5–15%), it slightly exceeds the 10% threshold suggested by the EASL guidelines for initiating center-level quality improvement measures [9,10]. This finding highlights the importance of continuous outcome monitoring and protocol optimization rather than indicating compromised donor safety.

The absence of Grade IV and V complications (life-threatening or fatal events) in our cohort is encouraging and supports the notion that donor hepatectomy can be performed with a low risk of severe morbidity under carefully controlled conditions. Nevertheless, these findings should be interpreted in the context of previously published registry-based and multicenter studies, which have consistently reported overall donor morbidity rates ranging between approximately 10% and 30%, with major complication rates generally below 10%.

In line with previous studies, biliary complications represented the most frequent source of postoperative morbidity in our cohort. This observation is not unexpected, given the anatomical complexity of the biliary tree, the presence of multiple ductal variants, and the need for precise transection during donor hepatectomy. Previous reports have consistently shown that bile leakage and biliary strictures account for a substantial proportion of donor morbidity, even in high-volume centers [25,26]. Importantly, most biliary complications can be effectively managed with minimally invasive techniques, such as endoscopic retrograde cholangiopancreatography or percutaneous drainage, thereby avoiding the need for reoperation [27,28]. Our findings support this pattern, as the majority of biliary complications in our cohort were successfully treated using non-surgical approaches.

Regarding donor-related factors, neither age nor BMI emerged as independent predictors of postoperative complications in multivariable analysis. These findings are consistent with previous studies suggesting that, within carefully selected donor populations, traditional demographic variables exert a limited influence on early postoperative outcomes. Notably, our BMI stratification was based on clinically relevant surgical risk thresholds rather than population-based classifications. This approach reflects real-world donor selection practices, in which both underweight and obese individuals may be considered at increased perioperative risk. Previous studies have suggested that extreme BMI values may influence wound healing, infection risk, and postoperative recovery; however, these associations appear to be attenuated when strict donor selection and perioperative protocols are applied [19,20,21].

Similarly, graft-related variables—including graft type, graft volume, and remnant liver ratio—were not independently associated with postoperative morbidity in our cohort. This finding suggests that adherence to established volumetric safety thresholds and anatomical principles may mitigate the potential impact of technical graft-related factors on early donor outcomes [29]. Previous studies have highlighted the remarkable regenerative capacity of the liver following donor hepatectomy, provided that an adequate remnant volume is preserved. Collectively, these observations underscore the importance of meticulous preoperative planning and comprehensive volumetric assessment rather than reliance on any single parameter.

Beyond volumetric considerations, hepatic parenchymal quality remains a fundamental determinant of donor safety in living donor liver transplantation. In our program, donors with cirrhosis or significant fibrosis are not accepted for donation, and moderate-to-severe hepatic steatosis is excluded. Therefore, the present cohort represents a carefully selected donor population with preserved parenchymal integrity, allowing volumetric parameters to be interpreted within a clinically safe and controlled framework.

From a forward-looking perspective, emerging regenerative and pharmacological strategies may further enhance donor safety in carefully selected settings. Experimental and translational studies suggest that stem cell-based regenerative approaches, including biomaterial-assisted delivery systems, may augment hepatic regeneration and improve parenchymal resilience. In parallel, pharmacological anti-steatotic and antifibrotic agents targeting metabolic dysfunction-associated steatotic liver disease are under active investigation and may contribute to optimizing hepatic parenchymal quality prior to donation [30,31].

In the broader hepatectomy literature, clinically recognized remnant liver volume thresholds vary according to hepatic parenchymal quality. While a remnant liver volume of approximately 20% may be acceptable for healthy livers, higher thresholds around 30% are often recommended in the presence of chemotherapy-associated liver injury, and up to 40% for fibrotic or cirrhotic livers to minimize the risk of postoperative liver dysfunction [22].

Although advanced donor age has traditionally been considered a relative risk factor, recent meta-analyses have demonstrated that elderly donors can safely undergo hepatectomy when appropriately selected [11]. Our findings are consistent with this evolving paradigm, as age alone did not independently predict postoperative morbidity.

An initially observed difference in crude complication rates between domestic and foreign donors did not persist after multivariable adjustment. In addition to clinical and procedural factors, psychosocial stressors associated with undergoing major surgery abroad—such as language barriers, limited social support, and challenges in postoperative follow-up—may contribute to differences observed in unadjusted analyses. However, these factors could not be directly measured in the present study and should therefore be interpreted as hypothesis-generating rather than causal explanations. This finding highlights the importance of cautious interpretation of unadjusted comparisons. Rather than reflecting a true biological or demographic effect, such differences are more likely attributable to residual confounding, unmeasured variables, or differences in perioperative support systems. Accordingly, donor nationality itself should not be regarded as a causal risk factor for postoperative morbidity. This observation should instead be considered hypothesis-generating and warrants further investigation in prospective, multicenter studies.

A major strength of the present study is the large sample size and the use of a standardized complication grading system. However, several limitations must be acknowledged. First, the retrospective and single-center design limits the generalizability of our findings. Second, the relatively low number of major complications reduces the statistical power to detect subtle associations, particularly in subgroup analyses. Third, our follow-up primarily focused on early postoperative outcomes. Although most donor complications occur during the early postoperative period, long-term surveillance is essential to capture late-onset complications, chronic symptoms, and psychosocial consequences that may not be evident during short-term follow-up [24]. Finally, residual confounding by unmeasured variables cannot be fully excluded.

This study provides a focused assessment of early postoperative outcomes in living liver donors managed under standardized protocols. The observed morbidity was predominantly of low grade, and no life-threatening complications or donor mortality were recorded. Biliary complications constituted the most frequent source of morbidity but were largely manageable with conservative or minimally invasive approaches. After multivariable adjustment, no donor- or graft-related variable, including nationality and BMI, was independently associated with postoperative morbidity, indicating that early complications are likely multifactorial rather than attributable to isolated demographic or anatomical factors. Given the retrospective design, single-center setting, strict donor selection, and limited follow-up duration, these findings should be interpreted as reflecting early postoperative safety only.

## 5. Conclusions

In this cohort of 502 living liver donors, early postoperative morbidity was low and no donor mortality was observed. These findings suggest the acceptable short-term safety of living donor hepatectomy under standardized protocols; however, conclusions regarding overall donor safety are limited by the study design, follow-up duration, and selection bias.

## Figures and Tables

**Table 1:** Demographic Data and Hospital Stay Durations.

Variable	*n*	Mean	Median	Min–Max
Age (years)	502	33.75	33.0	18–60
Weight (kg)	502	71.15	70.0	46.0–113.0
BMI	502	24.72	24.3	16.85–37.42
Turkish Citizen BMI	316	25.02	24.68	16.85–36.76
Foreign National BMI	186	24.21	23.77	16.98–37.42
Complication(+) LOS	58	6.58	5.7	3.0–21.0
Complication(−) LOS	444	4.98	5.0	2.5–9.0

LOS: length of stay; BMI: body mass index.

**Table 2:** Distribution of Complications and Treatment Approaches.

Clavien–Dindo	Complication	Treatment Method	*n*
Grade 1	Wound discharge	Dressing applied	7
	Gastritis	Endoscopy, medical treatment	1
	Diarrhea, pruritus, meteorism	Medical treatment	1, 1, 2
Grade 2	Pleural effusion	Medical treatment	3
	Bile leakage	Drain kept for longer period	2
	Subileus, ileus	Medical treatment	3, 1
	Wound infection	Dressing applied	4
	Effusion and atelectasis	Medical treatment	1
	Stool VRE positive, UTI	Medical treatment	1, 1
	Ileus, wound discharge	Medical treatment	1
	Pleural effusion, atelectasis	Medical treatment	1
	Pulmonary embolism, pneumonia	Medical treatment	1, 3
	Prolonged drain output	Drain kept for longer period	3
	Wound infection, pleural effusion	Dressing + medical treatment	1
Grade 3A	Bile leakage	ERCP and/or PCD performed	7
	Wound infection	Catheter inserted	1
	Atelectasis, pleural effusion	Pleurocentesis performed	1
	Atelectasis, bile leakage	PCD performed	1
	Intra-abdominal collection	Catheter inserted	1
	Iatrogenic bile duct injury	ERCP and PCD performed	1
	Pneumonia	Medical treatment	1
	Wound infection, pleural effusion	Pleurocentesis performed	1
Grade 3B	Incisional hernia	Herniorrhaphy performed	6

ERCP: endoscopic retrograde cholangiopancreatography; PCD: percutaneous catheter drainage; UTI: urinary tract infection; VRE: vancomycin-resistant enterococcus. Note: In the Clavien–Dindo classification, complications are graded based on the required treatment rather than the diagnosis itself; therefore, the same complication entity may appear in different grades depending on the therapeutic approach.

**Table 3:** Complication analysis by demographic, clinical, and surgical parameters with major/minor distribution and *p*-values.

Group	Total (*n*)	Complication Positive *n* (%)	*p*-Value	Minor Complication *n* (%)	*p*-Value	Major Complication *n* (%)	*p*-Value
BMI: <20	44	4 (9)	0.338	3 (6.8)	0.376	1 (2.3)	0.776
BMI: 21–29	397	50 (12.5)		33 (8.3)		17 (4.3)	
BMI: ≥30	61	4 (6.5)		2 (3.2)		2 (3.2)	
							
Right graft	407	49 (12.03)	0.759	30 (7.4)	0.748	19 (4.7)	0.232
Left graft	46	4 (8.69)		3 (6.5)		1 (2.2)	
Left Lateral graft	49	5 (10.2)		5 (10.2)		0 (0)	
							
Remnant < 30%	49	7 (14.3)	0.865	6 (12.2)	0.528	1 (2)	0.255
Remnant 30–39%	322	38 (11.8)		21 (6.5)		17 (5.3)	
Remnant 40–49%	34	4 (11.8)		3 (8.8)		1 (2.9)	
Remnant ≥ 50%	94	9 (9.9)		8 (8.5)		1 (1.1)	
							
Volume < 500 g	86	8 (9.3)	0.702	7 (8.1)	0.914	1 (1.2)	0.335
Volume 500–799 g	222	24 (10.8)		16 (7.2)		8 (3.6)	
Volume 800–999 g	154	20 (12.9)		11 (7.1)		9 (5.8)	
Volume ≥ 1000 g	39	6 (15.3)		4 (10.3)		2 (5.1)	
							
Turkish Citizen	316	44 (13.9)	0.0432	27 (8.5)	0.301	17 (5.4)	0.056
Foreign National	186	14 (7.5)		11 (5.9)		3 (1.6)	

**Table 4:** Multivariable logistic regression analysis of factors associated with postoperative complications.

Variable	aOR	95% CI	*p*-Value
Age	1.02	0.99–1.05	0.256
BMI	1.05	0.97–1.14	0.230
Remnant liver (%)	0.99	0.93–1.05	0.769
Graft volume	1.00	1.00–1.00	0.993
Male sex	1.07	0.60–1.90	0.815
Foreign nationality	0.03	0.00–1.12	0.058

Abbreviations: aOR, adjusted odds ratio; CI, confidence interval; BMI, body mass index.

## Data Availability

The data presented in this study are available on request from the corresponding author due to ethical and privacy restrictions.
